# Correlation of Transmission Properties with Glucose Concentration in a Graphene-Based Microwave Resonator

**DOI:** 10.3390/mi14122163

**Published:** 2023-11-27

**Authors:** Muhammad Yasir, Fabio Peinetti, Patrizia Savi

**Affiliations:** 1 Division of Microrobotics and Control Engineering, Department of Computing Science, University of Oldenburg, 26129 Oldenburg, Germany; 2 Department of Electronics and Telecommunications, Politecnico di Torino, 10129 Torino, Italy; fabio.peinetti@polito.it (F.P.); patrizia.savi@polito.it (P.S.)

**Keywords:** graphene, thick films, microstrip lines, lumped model, scattering parameters, ring resonator, functionalisation

## Abstract

Carbon-based materials, such as graphene, exhibit interesting physical properties and have been recently investigated in sensing applications. In this paper, a novel technique for glucose concentration correlation with the resonant frequency of a microwave resonator is performed. The resonator exploits the variation of the electrical properties of graphene at radio frequency (RF). The described approach is based on the variation in transmission coefficient resonating frequency of a microstrip ring resonator modified with a graphene film. The graphene film is doctor-bladed on the ring resonator and functionalised in order to detect glucose. When a drop with a given concentration is deposited on the graphene film, the resonance peak is shifted. The graphene film is modelled with a lumped element analysis. Several prototypes are realised on Rogers Kappa substrate and their transmission coefficient measured for different concentrations of glucose. Results show a good correlation between the frequency shift and the concentration applied on the film.

## 1. Introduction

Diabetes causes a number of health complications leading to the loss of precious human lives. These complications include hypoglycaemia, ketoacidosis, retinopathy, neuropathy, stroke, and heart diseases. This also results in an increased financial burden on health systems around the world. Diabetes is impacting an increasing number of people in Europe and the US. According to the World Health Organisation, there are about 60 million people with diabetes in the European region [1]. In the US, according to the Centre for Disease Control and Prevention, more than 37 million people suffer from diabetes [2]. While it is important to prevent the incidence of diabetes to protect the population, it is of utmost importance to actively monitor the blood glucose level of people suffering with diabetes in order to avoid health complications.

In order to provide multiple solutions to people with diabetes, there is a need to provide them with glucose detection solutions based on other body fluids. However, the change in blood glucose concentration is reflected in other bodily fluids (tears, saliva, and sweat) with a reduced concentration variation [3]. Therefore, there is a need to develop sensor systems capable of the targeted detection of glucose molecules at low concentrations.

The most common type of glucose sensors are electrochemical sensors based on a selective chemical reaction between enzymes and glucose [4,5]. An electrochemical glucose sensor is a device that transduces glucose concentration into a quantifiable electrical signal. In order to immobilise enzyme and glucose, substrates with certain characteristics, for example, a large surface area and easy functionalisation, are required. Nanomaterials are an attractive option since they possess these characteristics.

Graphene is one of the most commonly used nanomaterials. Graphene is a monolayer of carbon atoms, whose electronic, chemical, and mechanical properties have made it suitable for many cutting edge applications in the last few years [6]. Pure graphene cannot be directly used, but printable graphene-based inks can be manufactured by dispersing flakes into a proper solvent. Among the different printing techniques, the most used are chemical vapour deposition [7], drop casting [8,9], screen printing [10], and epitaxial growth [11].

Printed graphene film [10,12,13,14] can be used for many applications as flexible electronics [15], humidity sensors [16,17], electro-chemical sensors [18,19,20,21,22,23,24,25], bio-molecular and drugs detection, and monitoring [18].

For use in biological applications, graphene films are integrated as active components in biosensor systems [21,24,25]. These biosensor systems could be used for early cancer detection [26,27] as well as for chemical concentration detection in the body fluid such as sugar molecules [28] or glucose [29].

In [30], a similar surface immobilisation procedure was used to detect HRP (Horseradish Peroxidase) concentration, by measuring the frequency shift of the reflection coefficient of a microstrip antenna. The use of radio frequency (RF) biosensors based on passive and/or active devices and circuits has already been investigated [31,32,33]. These biosensors possess a high potential to modulate their sensitivity and selectivity using tailored chemical functionalisation to adsorb particular molecules. The performance of these biosensors can be enhanced by the introduction of nanomaterials. Multidisciplinary research capabilities are needed for the realisation of biosensors with high sensitivity and low concentration limits.

The goal of this paper is to use radio frequency signals to detect glucose concentration. The device used is a split ring resonator with a graphene film deposited in the split part of the ring. The film is appropriately functionalised for the detection of glucose. Varying concentrations of glucose are deposited on the functionalised film varying its impedance and resulting in a shift in the resonant frequency of the resonator. The levels of glucose concentration considered are low and correspond to the variation of glucose in bodily fluids other than blood (saliva, tears, sweat, etc.) [3,32]. A future sensor designed based on this principle will hence be a non-invasive glucose testing device. A preliminary form of this study has been published in [34], where the data are limited to simulations and measurements of a single device with only two concentration values. Here, an extensive study of the resonating device has been performed with more information about the film, surface functionalisation, the description of materials, circuit model, and more measurement points.

In Section 2, the ring resonators’ design and fabrication, and the enzyme immobilisation procedure over the film has been discussed. The realisation techniques of the graphene films and the scattering parameter measurements setup are introduced. In Section 3, full-wave simulations of the ring resonator without graphene film are discussed; a circuit model for the graphene film deposition is proposed and compared with full-wave simulations; drops with different glucose concentrations are deposited on the film and the corresponding calibration curves are derived. The variation of the resonant frequency versus glucose concentration over time has been analysed. In Section 4, some final considerations are drawn.

## 2. Materials and Methods

### 2.1. Prototype and Film Realization

The sensing element is a squared ring resonator with a gap. The ring resonator is simulated with the finite element-based simulation tool Ansys HFSS. The solution type used for the simulation is the driven modal type. Excitations are introduced at the end of the microstrip line feeding the resonating ring. A wave type excitation is used for both the ports. The dielectric material on which the resonator is placed is the Rogers Kappa 438. It has a nominal dielectric permittivity of $\epsilon_{r}=4.3$ and a loss tangent tanδ of 0.005 (@10GHz) at 2 GHz. The height of the dielectric substrate is 1.52 mm with a copper thickness of 35 mm. The gap is covered by a graphene film doctor-bladed with the help of a mask (diameter of 5 mm). The film thickness is around 500μm.

The film is composed of a binder, polyvinylidene fluoride (PVdF), in which graphene nanoplatelets are dispersed. The filler-to-binder ratio is 9:1. Graphene nanoplatelets were acquired from Nanoinnova, Spain. They have a surface area-to-weight ratio of $45\;m^{2}/g$ with a carbon content of 98.9 wt%. A material characterisation and detailed description of the graphene nanoplatelets can be found in [27]. The binder is first dispersed in a solvent, N-methyl-2-pyrrolidone (NMP); then the graphene nanoplateles are added. The filler (graphene), binder (PVdF), and solvent (NMP) are mixed overnight until a homogeneous mixture is obtained. Then, the mixture is doctor-bladed onto the mask. A drying process under hoof convection is performed for several days to obtain a film devoid of any solvent and humidity. Finally, the mask is removed and the film is ready to be functionalised.

The functionalisation of the film and immobilisation of the enzyme are carried out by the following procedure. It has been derived by adapting the procedure reported in [35] for silicon substrates. Glucose oxidase was immobilised on the graphene film with the help of carboxylic groups. Amide bonds are formed between the carboxyl groups of the carbon material of graphene and the amino groups of GOx. Drops of 1% *v*/*v* CPTES toluene solution in reflux solvent condition were deposited on the graphene film in experimental anhydrous conditions. This was followed by rinsing the film with toluene and drying it with nitrogen. Drops of $H_{2}{\mathrm{SO}}_{4}$ were subsequently deposited on the film in an Argon environment. This results in the hydrolysis of CN groups into COOH groups.

### 2.2. Scattering Parameters Measurements and Graphene Film Modeling

**Figure 1:**
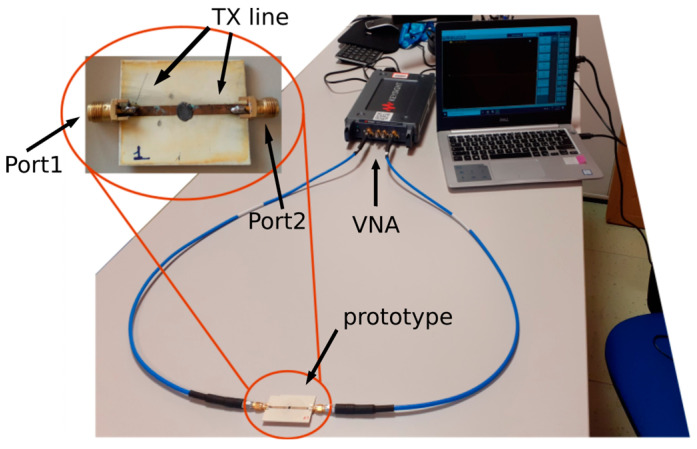
Scattering parameters measurement setup.

The scattering parameters of the transmission lines and ring resonators are measured with a a two-port USB vector analyser (VNA, P9371A) by Keysight, Santa Rosa, CA, USA (Figure 1).

The signal is generated and transmitted through a microstrip transmission line by a vector network analyser. The resultant reflections from the resonators are also measured by the VNA.

Measurements are performed in the 1 GHz to 5 GHz frequency range. Before the measurements, a two-port calibration process is performed with an automatic calibration kit.

A tuning process of the measured S-parameters leads to the derivation of the graphene film circuit model using AWR Microwave Office. Full-wave simulations with HFSS are performed to design the squared ring resonator. Full-wave simulations are also used to analyse the graphene film before and after the functionalisation procedure and to derive the values of the corresponding surface impedance.

## 3. Results

In this section, a ring resonator is first designed to resonate at 2.5 GHz without the graphene ink deposition. Some prototypes of this resonator are manufactured on Rogers Kappa 438 substrate. The graphene film is deposited and functionalised to detect glucose. The variation of the resonance frequency of the ring is measured for different glucose concentrations (5,10,20,30 mg/dL). Finally, calibration curves are provided for two samples and analysed in detail.

### 3.1. Ring Resonator Design

**Figure 2:**
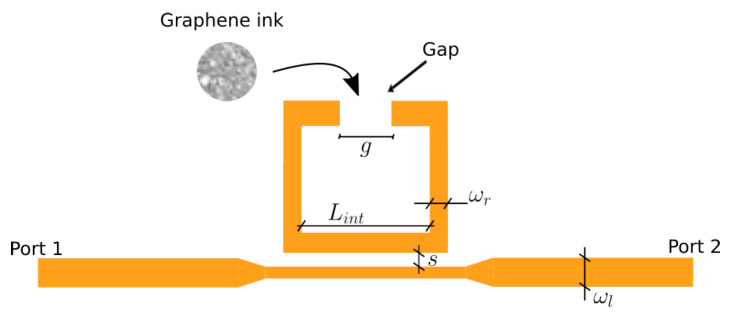
Geometry of the squared ring resonator.

The ring resonator is composed of a high impedance microstrip line fed split ring resonator, as shown in Figure 2. The resonator is fed by a microstrip line that has a characteristic impedance of 50Ω. This corresponds to a width, $w_{l}=2.9$ mm. The ring is coupled to the main line. The coupling distance between the ring and the line is s=0.2 mm. The internal size of the ring is $L_{int}=9$ mm. The width of the ring line is $w_{r}=1$ mm. The ring is split at one end where the graphene film is deposited. The width of the split area is $L_{g}=2$ mm.

**Figure 3:**
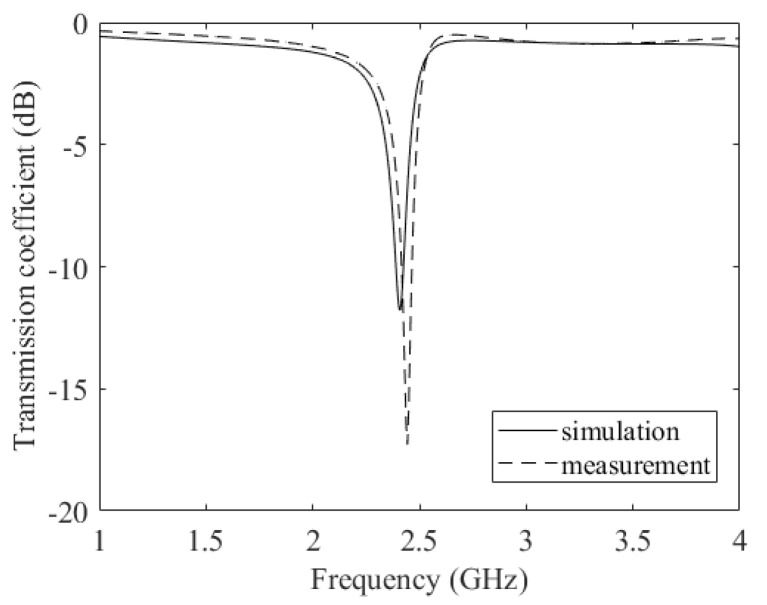
Simulated and measured transmission coefficient of the squared ring resonator.

The ring resonator is simulated with the help of the full-wave simulator Ansys HFSS on a Rogers 438 substrate (see solid line in Figure 3) and resonates at 2.5 GHz.

### 3.2. Graphene Film Circuit Model

In this section, a circuit model for the graphene film deposition is proposed and described in detail. By using circuit model parameters, a sheet impedance value for HFSS simulations is estimated.

**Figure 4:**
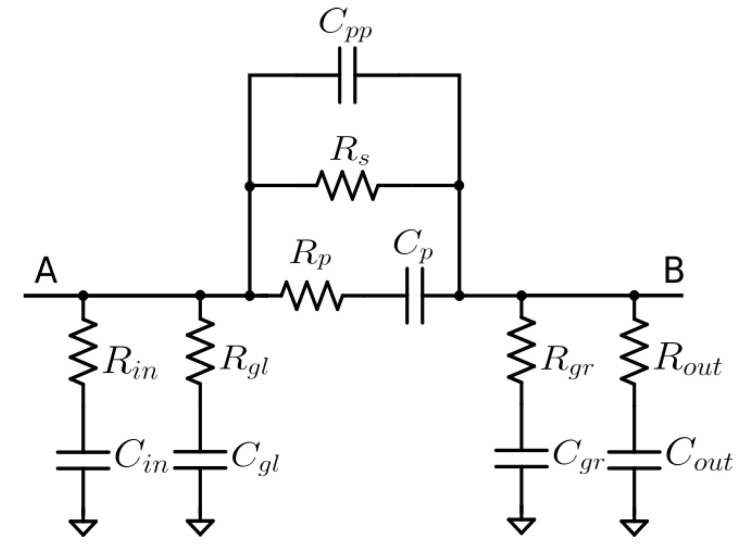
Graphene deposition equivalent circuit. Node A and B correspond to the deposition edges.

The circuit reported in Figure 4 can be suitably used to describe a graphene-filled gap of 2 mm. The model is chosen to be symmetrical, as is evinced by the measured S-parameters: $S_{11}\;=\;S_{22}$ and $S_{12}\;=\;S_{21}$.

With respect to a classic gap model [36], made up of capacitances, the series resistances model the losses in the dielectric at a high frequency (see $R_{gl},\;R_{gr}$, $R_{in},\;R_{out},\;R_{p}$). The chosen dielectric is Rogers Kappa 438, with a low loss tangent (tanδ=0.005 @10GHz), leading the resistive elements to be a few tens of Ohms. $R_{s}$ accounts for the sheet resistance, but is also the DC resistance (the only remaining element at zero frequency) between nodes A and B, and is linked to the percolative path in the deposition. As discussed in [37,38], an increase in graphene weight fraction makes $R_{s}$ lower as the percolative threshold falls. The described model takes into account both the graphene and the binder due to the impossibility of removing it. Even if the deposited slurry is a mixture of binder and filler, the sheet resistance is basically not influenced by the binder, which is non a conductive material, since the percentage of the binder in the filler is very small, compared to graphene. As for $C_{p}$, it represents the capacitance developed between the two lines, separated by the 2mm gap. On the other hand, $C_{gl}$ ($C_{gr}$) accounts for the capacitive effect due to the left (right) microstrip line and the ground plane (fringing field effect). Finally, $C_{pp}$ models the effect of nanoscale capacitors, for which graphene platelets are the conductive element, and the binder acts as a dielectric material.

The parameters reported in Table 1 are obtained by tuning the S-parameters measured from a microstrip line with the graphene deposition, as in Figure 1 (circled inset).

**Figure 5:**
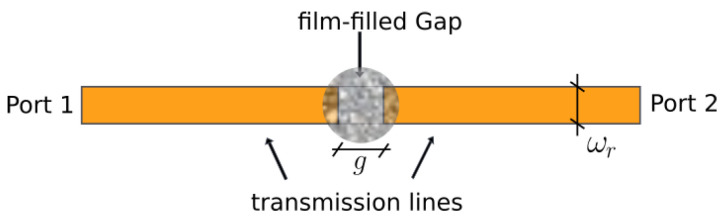
Microstrip line used for graphene model derivation.

A microstrip line 32 mm long and 3 mm wide, with a characteristic impedance of 50Ω, is divided into two equal parts by a 2 mm gap. The gap is filled by the graphene-based film as in Figure 5.

The width (W) of the transmission lines is 2.9 mm, so given a gap 2 mm wide (g) and the deposition diameter (d) of 5 mm, the aspect ratio (AR) is estimated to be:

(1) $$\mathrm{AR}=\frac{d}{g}=2.5$$

Consequently,

(2) $$Z_{sheet}=Z_{lumped}\times \mathrm{AR}$$

**Figure 6:**
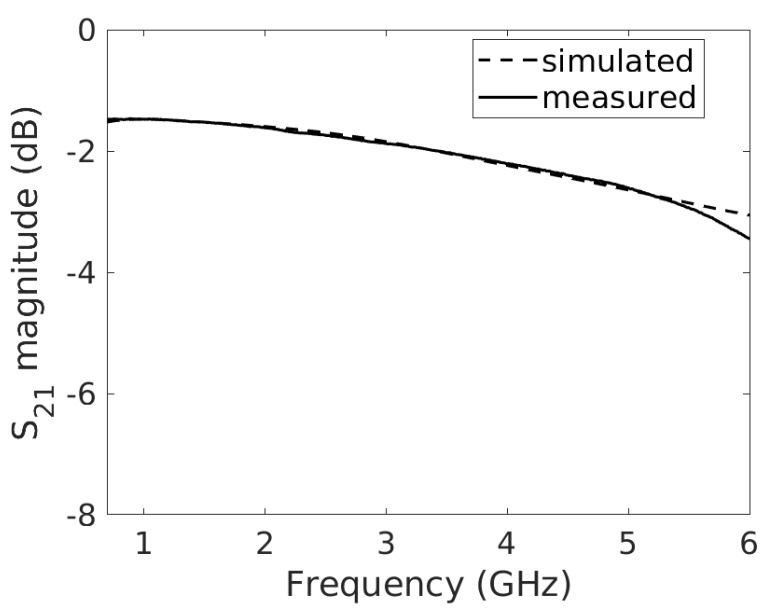
Graphene-filled gap: measured transmission coefficient (solid line) and circuit simulation (dashed line).

Simulations are performed in the 700 MHz to 6 GHz range and a comparison between measured and simulated parameters is reported in Figure 6 for the transmission coefficient. Even if not reported in Figure 4, two microstrip lines, connected to node A and B, respectively, were used in the simulations.

Figure 6 shows a good agreement between the measured values and the simulated values in the whole frequency range. A slight mismatch can be observed for frequencies above 5.5 GHz, due to the dielectric losses. The maximum variation between the measured and the simulated curves is less than 1dB for the transmission coefficient. As shown in [39], capacitances toward GND are mainly due to the binder. In the case of a line with a gap where the film is deposited, generally, a resistance variation of the film will result in an amplitude variation of the $S_{21}$, whereas a reactance variation will result in the phase variation of the $S_{21}$ [37].

### 3.3. Full-Wave Simulations of Graphene Film and Prototype Realization

**Figure 7:**
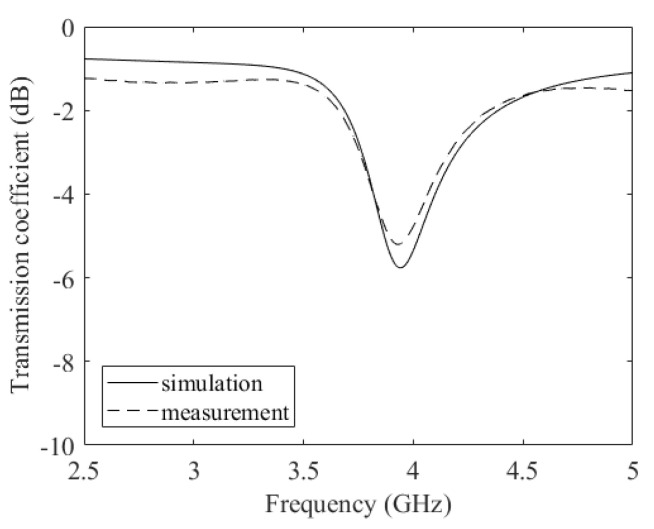
Ring resonator with graphene film. Measurements (dashed line) and simulation (solid line).

The circuital analysis performed in Section 3.2 resulted in an estimated value of the equivalent impedance of the film at the frequency of interest, i.e., 3.5 GHz. This value is (44−j6.11)Ω. This estimated value of the sheet resistance of the film was used as a starting point for the finite element simulations performed with the help of Ansys HFSS. Since the film deposited on the resonator has a diameter of 5 mm and the gap is 2 mm wide; therefore, the lumped impedance is increased by a factor of AR (see Equation (2)) in the finite element models. The resultant estimated value of the film was thus (111.5−j15.3)Ω/□. With this value, the resonance of the film did not exactly correspond to the measured value. This is because many parasitic impacts resulting from the film are taken in to account in the finite element simulations. Therefore, an optimisation of the finite element simulations was needed. For the FEM simulation values to correspond exactly to the measured values, a sheet impedance of (120−j10)Ω/□ was used. The resultant measured and simulated transmission coefficient of the resonator with the graphene film is shown in Figure 7.

**Figure 8:**
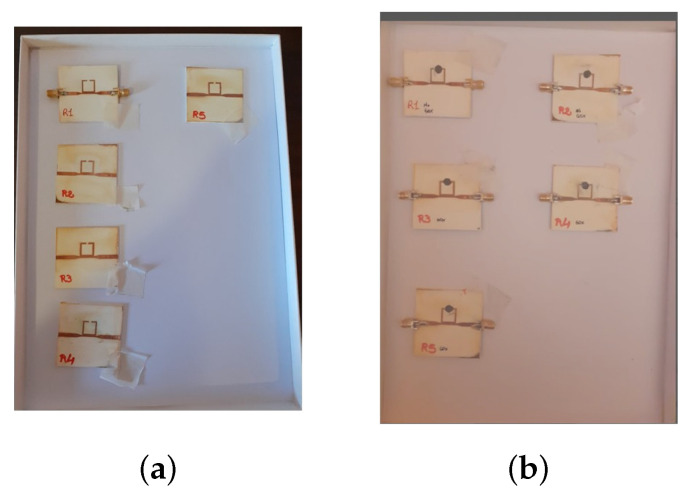
Pictures of the five ring resonator; in (**a**) those realized before the film deposition and in (**b**) after the deposition.

**Figure 9:**
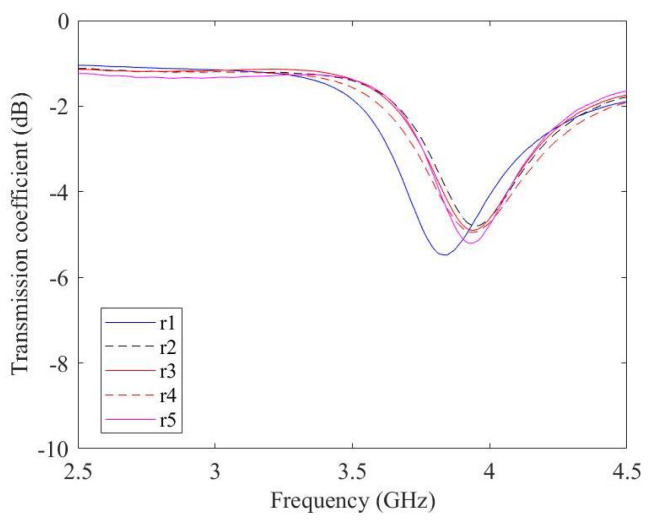
Measured transmission coefficient of the five-squared ring with graphene film.

Five prototypes of the resonator are fabricated and measured (Figure 8). Two of them were used for drop volume analysis; the other three were used for the experiments with glucose oxide. After the deposition of the graphene film across the gap, the resonators resonate at the frequency of 3.8 GHz as shown in Figure 9.

### 3.4. Film Functionalization

**Figure 10:**
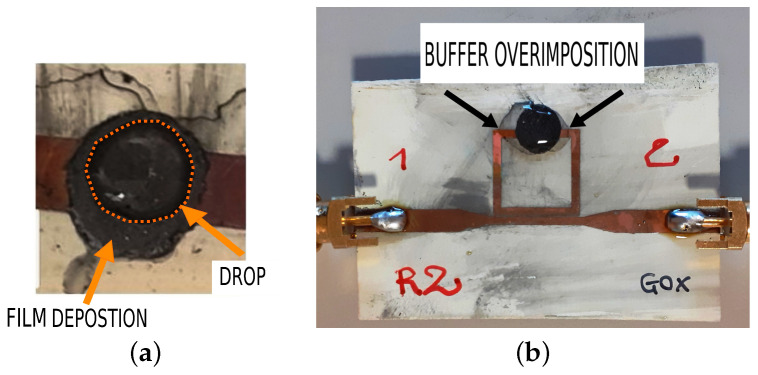
Drop dimensions analysis. (**a**) All the functionalised graphene is covered by the drop, so the drop interacts only with the film; (**b**) The drop covers both the graphene ink and the microstrip lines, directly connecting them. FILM to be replaced with ink.

The active area where the chemical process takes place is the film of graphene. The film is round in shape and with a diameter of 5mm. A large surface material, such as graphene, is exploited to accelerate the electron transfer from glucose oxidase toward the substrate and to promote the electrocatalytic performance of some molecules such as H${}_{2}$O${}_{2}$ [40]. The graphene film should be functionalised in order to be selective to a specific molecule. In this case, the surface of the film is functionalised with glucose oxidase with a standard procedure presented in [41]. On the functionalised surface, a drop of glucose and buffer solution (phosphate citrate, pH 7.2) of different concentrations is deposited with a micro pipette. The glucose drop is composed of 50mM of buffer, 0.3mM of TMB, and 10% ethanol for the drop with 5mg/dL concentration. The drop should be of a specific volume (30μL) in order to cover the surface (Figure 10a) but not in too much excess (over 60μL) so that it does not fall off or contact the edges of the stub (Figure 10b).

**Figure 11:**
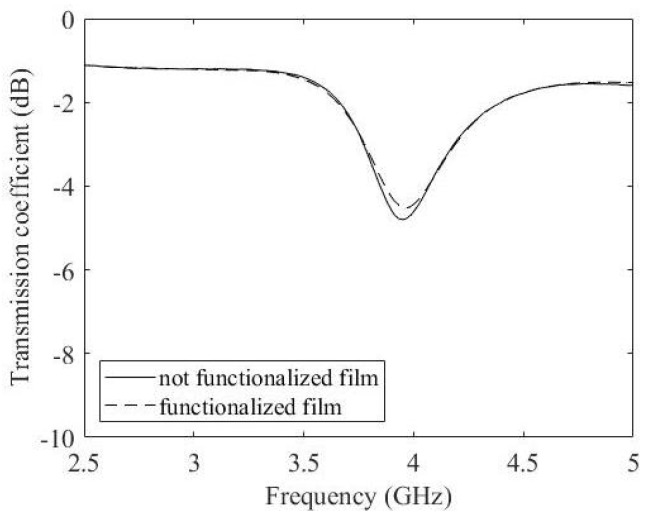
Measured transmission coefficient with not functionalised film (solid line) and functionalised film (dashed line).

Without the graphene film, the ring resonator resonates at 2.5 GHz (Figure 3). After the introduction of the film, the ring resonates at around 4 GHz (Figure 4). Note that there is a variation in the mismatch due to the conductive behaviour of the film. The resonator is measured before and after the functionalisation of the graphene film. With the introduction of the graphene film, the transmission coefficient is slightly altered without significant variations (Figure 11).

**Figure 12:**
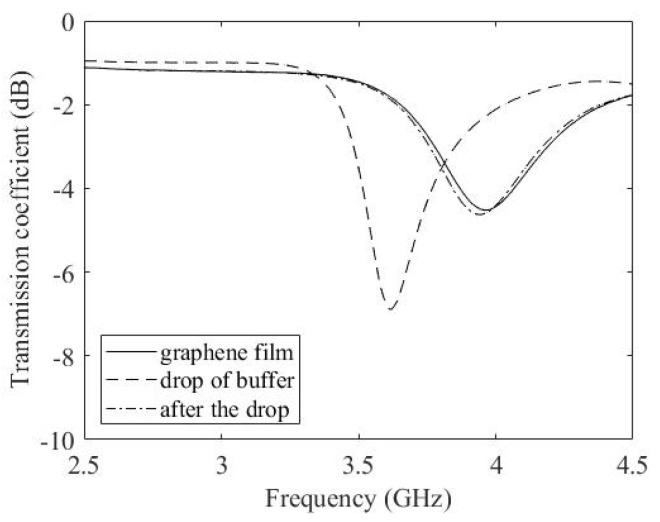
Measured transmission coefficient with and without a drop of buffer.

The presence of the buffer drop over the film shifts the resonant frequency from 4 GHz to 3.5 GHz as shown in Figure 12. In order to exclude the impact of the sole presence of the drop over the film, a drop of buffer is deposited over the film. We considered this frequency as the reference point for the glucose concentration experiment that follows.

**Figure 13:**
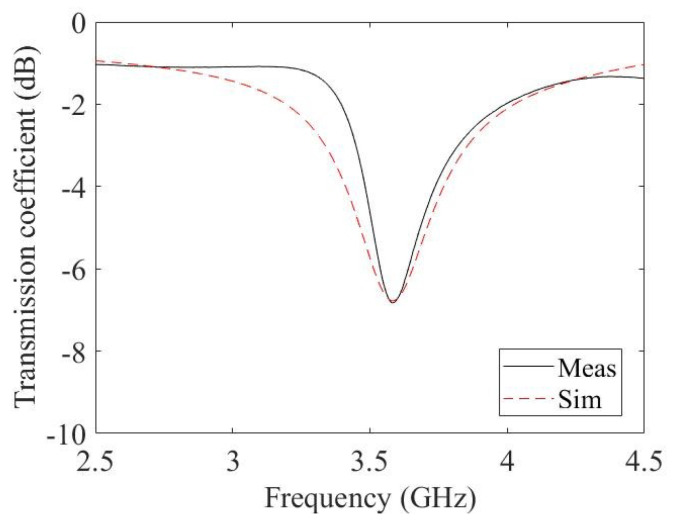
Ring resonator with functionalised glucose oxidase graphene film and a drop of buffer. Measurements (solid line) and simulation (dashed line).

The resultant measured and simulated transmission coefficient of the resonator with the drop of buffer is shown in Figure 13. The full-wave simulations were obtained with a surface impedance of (50+j200)Ω/□. The presence of the drop of buffer drastically changes the imaginary part of the sheet impedance. This is due to an increase in the conductivity of the sheet, which tends to reduce its capacitance.

### 3.5. Glucose Sensor Measuring Range

A number of different bodily fluids, including saliva, sweat, and tears, can be used to estimate the concentration of glucose in blood. The concentration of the glucose in each of the bodily fluids is different. The range of detection of the sensor depends on the type of the bodily fluid taken into account. Some relevant recent works are reported in Table 2.

**Figure 14:**
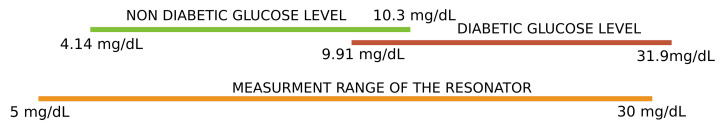
Glucose concentration in saliva compared to the range analysed in this work (orange curve).

As indicated in [3], a non-diabetic person’s salivary glucose concentration is between 4.1 mg/dL and 10.3 mg/dL, while in the case of diabetic samples, the range is shifted to between 9.9 mg/dL and 31.9 mg/dL. Also, ref. [49] confirms that salivary glucose levels in diabetic subjects lie in the range from 10.00mg/dL to 32.00 mg/dL. Differently, non-diabetic people’s levels span between 4.30 mg/dL and 12.90 mg/dL. Since the target of this work is to consider the detection of glucose in saliva, a range of glucose concentration of 5 mg/dL–30 mg/dL was chosen (Table 2). The dimension of the sensor, the size of the film, and the type of functionalisation are appropriately selected to cover this range (Figure 14).

### 3.6. Analysis of Different Glucose Concentrations

**Figure 15:**
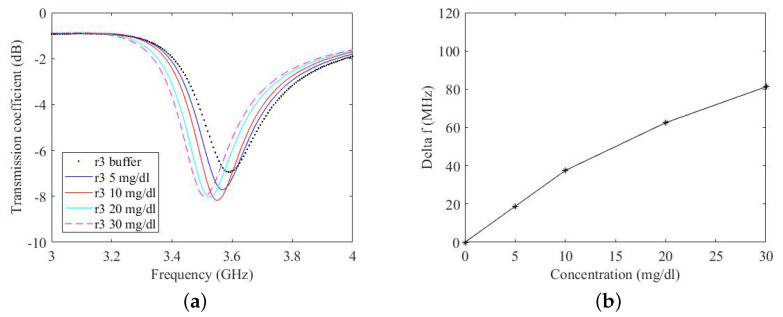
(**a**) Measured transmission coefficient for different glucose concentrations on prototype r3. (**b**) Calibration curve.

**Figure 16:**
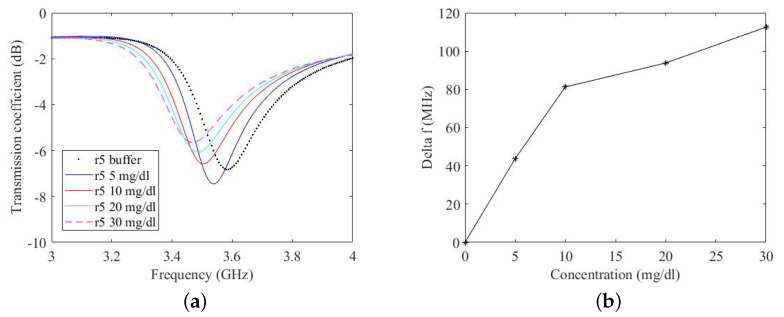
(**a**) Measured transmission coefficient for different glucose concentrations on prototype r5. (**b**) Calibration curve.

Each ring resonator is connected to an NA and two-port measurements of the transmission coefficient ($S_{21}$) are performed in the frequency band 1 GHz–5 GHz. As the aim of this work is to quantify the variation of concentration of glucose in terms of the frequency shift of the ring resonator, drops with 20μL volume of different concentrations from 0 to 30 mg/dL are deposited on the functionalised film. The introduction of various concentrations of glucose over the film varies its impedance. This change in impedance results in a shift of the resonant frequency of the resonator. The resulting transmission coefficients of the ring resonator are shown in Figure 15a for prototype r3 and in Figure 16a for prototype r5. It can be seen from (Figure 15a) that, with a concentration of 10 mg/dL, the ring resonates at a frequency of 3.56 GHz. Increasing the concentration to 30mg/dL shifts the resonant frequency to 3.52 GHz, corresponding to a shift in frequency of 40 MHz. Similarly, there is a shift in frequency of 20 MHz for the prototype r5 (in the 10 mg/dL–30 mg/dL concentration interval). Note that every time a drop of a different concentration is deposited on the film, the film is washed with a buffer solution. This shifts the resonant frequency of the ring back to its initial value.

In Figure 15b and Figure 16b, the measured frequency shift is reported as a function of the glucose concentration. For both prototypes, there is a change in the slope of the response at 10 mg/dL. The shift in frequency is greater in the case of prototype r5.

There is a difference in the frequency shift of the prototype r3 and the prototype r5. For example, the frequency shift of the prototype r3 with a concentration of 5 mg/dL is 20 MHz whereas, at the same concentration, the frequency shift of the prototype r5 is 42 MHz. As the glucose concentration increases, the difference in the frequency shift of the two prototypes is reduced. The frequency shift of the prototype r3 at a concentration of 30 mg/dL is 80MHz and that of the prototype r5 at the same concentration is 110MHz. This shows that the accuracy of the ring resonator increases with increased concentration of glucose. It is pertinent to mention that these concentrations are extreme, even if compared to standard glucose concentrations in body and those measured by commercial glucose detection devices. This phenomenon of decreased accuracy in terms of frequency shift at a very low concentration will be studied in a future article.

**Figure 17:**
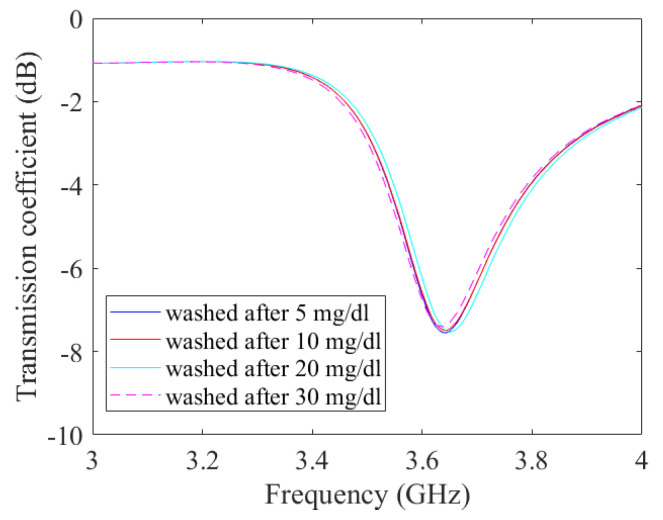
Measured transmission coefficient after washing prototype r5 for different glucose concentrations.

In order to verify the stability of the functionalised graphene film and exclude the impact of the previous concentration used, the transmission coefficient is measured after each washing procedure. The results are shown in Figure 17 for r5. It can be seen that the original condition is reached again after each washing step.

### 3.7. Performance of the Ring Resonator over Time

It is important to validate the performance of the ring resonator over time for different values of glucose concentration. The reaction between glucose and GOx, which takes place on the graphene surface, is not a kinetic reaction. It is important to evaluate how fast the reaction actually takes place.

**Figure 18:**
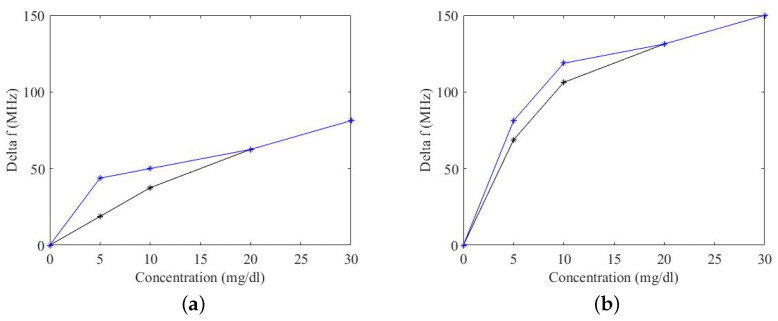
Calibration curves comparison over time. Blue curve represents the frequency variation at t1, while the black curves represent the frequency variation at t0. (**a**) Prototype r3 and (**b**) Prototype r5.

To this aim, the scattering parameters of the ring resonators were measured for different glucose concentrations at $t_{0}$ (reference measurement) and $t_{1}=60\;$s, see Figure 18b. In all cases, there is a variation in the resonant frequency of the resonators with a variation in the glucose concentration. For lower values of glucose concentration, there is a slight increase in frequency shift (Δf) over time, from $t_{0}$ to $t_{1}$. This increase in the frequency shift is not present for glucose concentration above 20mg/dL. This could be due to the depletion of the available GOx on the film surface.

## 4. Conclusions

Electrical detection of the concentration of glucose has been performed with the help of a passive two-port device consisting of a squared ring resonator working at microwave frequency. The active part of the resonator is a graphene-based film, fabricated and doctor-bladed on a designated spot on the ring resonator. The film is selectively functionalised for the detection of glucose molecules. Drops with different concentrations of glucose are deposited on the functionalised film, which result in a variation of the impedance of the film. The variation of the impedance of the film results in a shift of the resonant frequency of the ring. This is read in the transmission scattering parameter of the microstrip line feeding the ring.

The presence of other body analytes could potentially alter the entire response (e.g., by shifting the resonant peak to a different frequency or changing the amplitude) but, due to the functionalisation of the graphene surface, only glucose molecules attach to the surface of the film causing the frequency shift.

An evaluation of performance over time is carried out. For low glucose concentrations (<20 mg/dL), slightly higher differences in the resonant peak shift take place when a larger reaction time is provided, whereas for high glucose concentration (≥20 mg/dL), the frequency shift over time remains constant.

Equivalent circuit models of the graphene film are computed, followed by full-wave simulations and the fabrication and measurement of a number of prototypes. A maximum frequency shift of 110 MHz was obtained for a glucose concentration variation of 30 mg/dL. This is a first step towards the realisation of a non-invasive glucose measuring device.

## Figures and Tables

**Table 1:** Graphene deposition electrical model parameters.

$R_{s}$	$C_{\mathrm{pp}}$	$R_{p}$	$C_{p}$	$R_{\mathrm{gl}}$	$C_{\mathrm{gl}}$	$R_{\mathrm{gr}}$	$C_{\mathrm{gr}}$	$R_{\mathrm{in}}$	$C_{\mathrm{in}}$	$R_{\mathrm{out}}$	$C_{\mathrm{out}}$
(Ω)	(pF)	(Ω)	(pF)	(Ω)	(pF)	(Ω)	(pF)	(Ω)	(pF)	(Ω)	(pF)
46	0.14	18	12	276	0.06	276	0.06	90	0.15	90	0.15

**Table 2:** Enzymatic and non-enzymatic glucose concentration sensors comparison.

Data from	Measured Physiological Fluid	Type of Sensor	Min mg/dL	Max mg/dL
[42]	blood and saliva	Enzymatic	180 $\times \;10^{-3}$	180
[43]	sweat	Enzymatic	1.4	252
[44]	-	Non-enzymatic	1.2	3.6
[45]	blood	Enzymatic	18	72
[46]	saliva	Enzymatic	0.57	108
[47]	tears	Non-enzymatic	37.8 $\times \;10^{-3}$	360
[48]	-	Enzymatic	54 $\times \;10^{-3}$	720 $\times \;10^{-3}$

## Data Availability

The data that support the findings of this study are available from the corresponding author upon request.
